# Effects of guava pomace combined with gut modulators on growth performance, cecal histomorphology and gene expression in rabbits

**DOI:** 10.1038/s41598-026-57446-w

**Published:** 2026-06-18

**Authors:** Hadeel S. El-Qaliouby, Fathy Abdel-Fattah, Abeer Gamal Zaki, Ahmed I. Abo-Ahmed, Ayman H. Abd El-Aziz

**Affiliations:** 1 Department of Animal Wealth Development, Animal and poultry production, Faculty of Veterinary Medicine, Benha University, Moshtohor, Toukh, 13736 Egypt; 2 Department of Nutrition and Clinical Nutrition, Faculty of Veterinary Medicine, Benha University, Moshtohor, Toukh, Qalyubia, 13736 Egypt; 3 Biotechnology Department, Animal Health Research Institute (AHRI), ARC, P.O: 12618, Giza, Egypt; 4 Department of Anatomy and Embryology, Faculty of Veterinary Medicine, Benha University, Moshtohor, Toukh, 13736 Egypt; 5 Department of Animal Wealth Development, Faculty of Veterinary Medicine, Damanhour University, Damanhour, 22511 Egypt

**Keywords:** Guava pomace, Organic acids, Rabbit performance, Cecal histomorphology, Immune gene expression, Biotechnology, Immunology, Physiology, Zoology

## Abstract

Sustainable intensification of rabbit production necessitates the exploration of non-conventional feed resources to mitigate the rising costs and limited availability of conventional ingredients. The present study evaluated the effects of dietary inclusion of dried guava pomace (DGP), alone or combined with a prebiotic source of mannan-oligosaccharides and β-glucan or an organic acid blend, on growth performance, selected hemato-biochemical parameters, cecal histomorphology and some growth and immune-related gene expression in healthy New Zealand White growing, 28-day old, male rabbits. Compared with the control diet, the DGP-based diet presented significant improvements in final weight, final weight gain, feed intake, and feed conversion ratio as well as performance index (*P* < 0.01). Furthermore, fortification of diets with an organic acid blend plus DGP improved selected hematological indices, with no signs of liver or kidney impairment. Examination of cecal histomorphology showed increased villus height and preserved mucosal integrity in rabbits fed DGP-based diets. Gene expression analysis revealed increased muscle insulin-like growth factor-1 (IGF-1) in supplemented groups, whereas expression of inflammatory markers (TNF-α and IL-1β) showed tissue-specific response. Cecal inflammatory gene expression was not significantly affected. These findings indicate that DGP can be used in rabbit diets, alone or with functional additives, as a promising strategy for supporting rabbit health and productivity. Further study is needed to evaluate optimal inclusion levels, characterize bioactive components, assess long-term physiological effects, gut microbiota outcomes, and assess economic viability under commercial production conditions.

## Background

Rabbit farming has garnered considerable attention recently due to their fast growth and high reproductive rates compared to broiler chickens^1,2^. Rabbit meat is highly esteemed for its nutritional qualities, including a high protein content and low levels of fat and cholesterol, and favorable proportion of unsaturated to saturated fatty acids, making it an excellent source of lean animal protein for human consumption^3,4^. In 2017, data from the Food and Agriculture Organization (FAO) placed Egypt is among the leading rabbit-producing countries worldwide and responsible for approximately 3.8% of global output and an estimated 62,262 metric tons^5^.

In addition to meat production, rabbits provide fur and leather and benefits from the rapid reproductive rate and ability of the species to thrive on inexpensive diets made from forages and agricultural byproducts^6^. Among commonly used breeds, the New Zealand White (NZW) rabbit breed is highly valuable for meat production owing to its effective feed conversion and superior skeletal framework^7^.

Despite these advantages, rabbits particularly during the post-weaning stage are highly vulnerable to gastrointestinal disorders, which can impair growth performance and increase morbidity and mortality. For decades, antibiotics have been added prophylactically to feed as growth promoters to reduce the abovementioned complications^8,9^. However, increasing concerns regarding antimicrobial resistance and food safety have led to strict regulations and bans on antibiotic use, particularly within the European Union^10,11^. These restrictions have intensified research efforts toward natural and safe dietary alternatives, including prebiotics, probiotics, organic acids and enzymes and plant-derived bioactive compounds^12^.

Agro-industrial fruit byproducts have recently gained attention as functional feed ingredients due to their content of dietary fiber and antioxidant compounds. Guava (*Psidium guajava* Linn.) is extensively planted in tropical and subtropical regions of several countries. It is a member of the *Myrtaceae* family and is classified within the genus Psidium, and the species is Guajava, often known as Guava^13^. The fruit is distinguished by its succulent pericarp and pulp, which contains countless small seeds. It has multiple applications in human nutrition, including drinks, purees, jams, canned slices, syrup concentrates, and juices. In 2013, the global production of mangoes, mangosteens, and guavas exceeded 43 million tons, with Egypt contributing approximately 834,543 tons to this total^5^. Guava is a substantial source of numerous minerals, including vitamin C, which exceeds that of fresh orange juice, as well as considerable amounts of vitamin A, calcium, phosphorus, riboflavin, pantothenic acid, thiamin, pectin, niacin, and total free phenol. These components are known for their antioxidant effects^14^.

Prebiotics such as mannan-oligosaccharides, fructo-oligosaccharides, *saccharomyces cerevisiae* and β-glucan, derived from yeast cell walls, are commonly utilized in diets as a potential alternative to antibiotics to support gut health and immune response in rabbits and poultry^3,15–17^. Similarly, organic acids such as lactic, citric, fumaric and formic acids are used with their salts as alternatives to antibiotic growth promoters, where they enhance nutrient digestibility, regulate gastrointestinal pH, enhance proteolytic enzyme activity, and contribute to microbial balance within the gut ecosystem^18^. Aljumaah^19^ also found that dietary organic acids can alter short-chain fatty acids in the gut (butyrate and acetate), which impacts favorably on gut health and performance in broiler chickens challenged with *Salmonella Typhimurium*.

Although guava and its byproducts and individual gut-modulating additives have been studied separately, limited information is available regarding their combined uses in rabbit nutrition. Data are scarce on how guava pomace, when included alone or in combination with prebiotic or organic acid-based supplements, influences growth performance, hemato-biochemical parameters, intestinal morphology, and the expression of genes associated with growth and local immune regulation. Therefore, the current study aimed to evaluate of dietary inclusion of dried guava pomace alone or in combination with a prebiotic source of mannan-oligosaccharides and β-glucans or with an organic acid blend, on growth performance, selected hemato-biochemical parameters, cecal histomorphology and the expression of some key growth and immunity-related genes (IGF-1, TNF-α and IL-1β) in NZW rabbits. This work provides a novel insight into the value of combining agro-industrial byproducts with functional feed additives as a sustainable strategy for enhancing rabbit health and production efficiency.

## Materials and methods

### Study location and ethical considerations

The study was conducted at the Laboratory Animal Research Unit, Faculty of Veterinary Medicine, Benha University, Egypt, at the period from 7th January until 10th march, 2023. Ethical approval was granted by the Ethics Committee of Scientific Research, Faculty of Veterinary Medicine, Benha University, Egypt. All animal procedures were carried out according to the Institutional Animal Care and Use Committee guidelines (IACUC) with an approval number [BUFVTM 44-09-23]. Numerous precautions were taken to ensure that the animals did not suffer during the experiments. This study is reported in accordance with the ARRIVE guidelines (https://arriveguidelines.org).

### Animal acquisition and housing

Forty-eight weaned NZW male rabbits, 28 days of age with an initial body weight 436.15 ± 12.13 g, were purchased from a commercial rabbit farm in Qalyubia Governorate, Egypt. Upon arrival, rabbits were clinically examined and allowed to acclimatize under experimental conditions.

The weaned rabbits were divided randomly into four experimental dietary groups, with 12 rabbits per group (3 replicates). Each pair of rabbits was kept separately in a 40 × 60 × 35 cm (width × length × height) wire mesh cage with 24 cages in total. The cages were equipped with hopper feeders and nipple drinkers for free access to water. The rabbits were bred at controlled temperatures ranging from 14 to 20 °C and a relative humidity ranging from 59 to 65%. The only source of light was natural daylight.

### Diet formulation and experimental design

Fresh guava pomace (GP) was purchased as a wet byproduct of guava juice manufacture from a commercial juice-processing facility (Vitrac^®^ Company, Qaha, Egypt) and it was dried in an oven at 60 °C for 2 days. Then it was crushed, well mixed and stored in a well-ventilated place. The proximate chemical analysis of dried guava pomace (DGP) samples were performed according to A.O.A.C^20^.

To meet the dietary needs of rabbits, the NRC guidelines^21^ were followed in the formulation of the basal experimental diet (Table 1). Ingredient inclusion levels were adjusted to achieve similar concentration of crude protein (approximately 18%), crude fiber (approximately 15–16.5%) and digestible energy (approximately 2530–2600 kcal/kg DM) across treatments, which allow for incorporation of 20% DGP in the experimental diets.

**Table 1:** Feed composition and chemical analysis of the experimental diets (% DM) fed to growing rabbits.

Item	Control	T1	T2	T3
**Feed ingredients (% as fed)**				
Guava Pomace (GP)	0.00	20.00	20.00	20.00
Yellow corn	35.00	25.00	25.00	25.00
Soybean meal (44% CP)	31.20	24.60	24.60	24.60
Wheat bran	0.00	24.00	23.90	23.90
Fennel straw	28.00	0.00	0.00	0.00
Molasses	2.00	2.00	2.00	2.00
Prebiotic source (MOS & β-glucan)	0.00	0.00	0.10	0.00
Organic acids blend	0.00	0.00	0.00	0.10
DL-Methionine	0.40	0.40	0.40	0.40
L-Lysine	0.00	0.10	0.10	0.10
Vitamin-mineral premix^1^	0.50	0.50	0.50	0.50
Salt	0.50	0.50	0.50	0.50
Limestone	1.10	2.90	2.90	2.90
Dicalcium phosphate	1.30	0.00	0.00	0.00
Total	**100.00**	**100.00**	**100.00**	**100.00**
**Chemical analysis (% DM)**				
Crude protein	18.00	18.03	18.01	18.01
Crude fiber	14.86	16.46	16.45	16.45
Neutral detergent fiber (NDF) ^2^	38.68	39.73	39.73	39.73
Acid detergent fiber (ADF) ^3^	22.98	24.44	24.43	24.43
Calcium	1.20	1.21	1.21	1.21
Total phosphorus	0.61	0.65	0.65	0.65
Lysine	0.91	0.81	0.81	0.81
Methionine + Cystine	0.68	0.61	0.61	0.61
Digestible energy (kcal/kg) ^4^	2529.40	2596.29	2593.80	2593.80

^1^Purchased by Onyx Veta for veterinary products, Mehalla Road, Tanta city, Egypt. Mineral and vitamin premix composition (per kg of complete feed): vitamin A = 4,000,000 IU, D3 = 666,666.67 IU, E = 3,333.33 mg, K3 = 333.333 mg, B1 = 333.333 mg, B2 = 1,666.67 mg, B6 = 500 mg, B12 = 3.33 mg, niacin = 10,000 mg, biotin = 16.67 mg, folic acid = 333.33 mg, pantothenic acid = 3,333.33 mg, choline chloride = 166,666.67 mg, zinc = 16,666.67 mg, manganese = 20,000 mg, iron = 10,000 mg, copper = 3,333.33 mg, iodine = 333.33 mg, selenium = 33.33 mg, cobalt = 33.33 mg, and calcium carbonate to 1 kg. **[**^2^Neutral detergent fiber (NDF%) = 28.924 + (0.657 × CF %), ^3^Acid detergent fiber (ADF%) = 9.432+ (0.912 × CF %)^22^**]**. ^4^Digestible energy (DE) was calculated according to values given in the feed composition tables of NRC^21^.

The dietary treatments consisted of:

- CON (control): commercial basal diet without additives (consists mainly of soybean meal, corn, fennel straw and molasses).
- T1: basal diet supplemented with 20% DGP.
- T2: basal diet supplemented with 20% DGP and 1.0% prebiotic source of MOS and β-glucans (Y-MOS^®^, Nutrex, Olen, Belgium).
- T3: basal diet supplemented with 20% DGP and1.0% of a mixture of organic acids, propionate, binder and additional supportive components (Fylax forte^®^, Selko, Amersfoort, Netherlands).

The idea behind the percentage of guava pomace used in this experiment is from previous research papers which concluded that guava waste can be utilized in the diet at a level of 20% without adverse effect on performance and health status in a previous research on growing rabbits^23,24^. While other additives were supplied according to the manufacturer’s recommendations. All diets were supplemented with standardized salts, vitamins, and minerals. A specialized feed mill was used to prepare pelleted diets (4 mm diameter, 9 mm length). The rabbits were allowed to adapt to their diets for 1 week before they had access to ad libitum until the end of the experiment, when they were 98 d of age.

### Growth performance indices

The rabbits were weekly weighed individually in the morning throughout the experimental period to calculate the final body weight (FBW), final weight gain (FWG), average daily gain (ADG), daily feed intake (DFI) as well as total feed intake (TFI), and feed conversion ratio (FCR) according to Abd El-Aziz et al.^3^. where the cage was used as the experimental unit for calculating FI and FCR. In addition, performance index (PI), which was calculated according to the formula of North^25^.

### Assessment of biochemical and hematological parameters

At the conclusion of the trial (98th day), the rabbits were humanely euthanized using an intravenous overdose of sodium pentobarbital (150 mg/kg BW) following anesthesia with ketamine (35 mg/kg BW, intramuscular) and xylazine (5 mg/kg BW, intramuscular). Blood samples (*n* = 6 samples per treatment group) were collected from the jugular vein (about 5 mL per rabbit) using sterile tubes. For hematological analysis, blood was collected in EDTA-coated tubes and analyzed immediately. For biochemical analysis, blood samples were collected in plain tube, allowed to clot at room temperature, and plasma was separated by centrifugation at 4,000 rpm for 15 min using a Sigma laboratory centrifuge (Model 1–14, Part No. 10014, Serial No. 163123, Sigma Laborzentrifugen GmbH, Osterode am Harz, Germany) and stored at − 20 °C until analyses were performed. Liver enzymes, including alanine aminotransferase (ALT; IFCC Fluid (5 + 1): Catalogue No. GF05000060, Lot/Edition No. 04/2013) and aspartate aminotransferase (AST; IFCC Fluid (4 + 1): Catalogue No. GF09000050, Lot/Edition No. 09/2017) were assessed by the kinetic IFCC method (without pyridoxal phosphate activation) using Centronic GmbH kits (Germany) according to previously described methods^26^, whereas kidney function was assessed on the basis of plasma creatinine (CR; using the kinetic Jaffe method without deproteinization following the manufacturer’s instructions (Centronic GmbH, Germany; Creatinine Jaffe Kinetic Fluid (1 + 1), Catalogue No. CF11000100) and urea levels was determined by the urease–colorimetric method using the Urea/BUN Colorimetric Kit (Salucea BV, The Netherlands; Cat. No. NS318001) according to^27,28^. Furthermore, albumin (ALB) was determined colorimetrically using the Albumin Fluid Mono Kit (Bromocresol Green Method) (Centronic GmbH, Germany; Cat. No. AF01000050) and total protein (TP) was analyzed by the Biuret colorimetric method using the Total Protein Biuret Reagent Kit (Spectrum Diagnostics, Egyptian Company for Biotechnology-Spectrum Diagnostics, Cairo, Egypt; Cat. No. 310001) following the method of^29,30^. Globulin (GLOB) was measured by the difference between TP and ALB^31^. Hematological assessments included red blood cells (RBCs) and white blood cells (WBCs) count, as well as differential leukocyte counts, and packed cell volume (PCV) and hemoglobin (Hb) levels were measured according to the procedures outlined by Feldman et al.^32^.

### Quantitative gene expression analysis of IGF-1, IL-1β, and TNF-α Using qRT-PCR

After dissection, 1 g of both cecal and muscle tissues were packed into a 2 ml Eppendorf tubes, directly frozen in liquid nitrogen and stored at −70 C for RNA isolation. Then, to assess gene expression in response to dietary treatment, total RNA was extracted from both the cecal and muscle tissues of the rabbits (*n* = 3 per treatment group) via the Easy-Red reagent (iNtRON Biotechnology, Seongnam-si, South Korea) following the manufacturer’s protocol. The quality and quantity of the extracted RNA were verified via agarose gel electrophoresis, and visualization was performed with ethidium bromide.

First-strand complementary DNA (cDNA) was synthesized via TOPscript™ RT Dry MIX (Thermo Scientific, Waltham, MA, USA) following the manufacturer’s instructions. Quantitative real-time PCR (qRT‒PCR) was carried out on a total of 96 samples, to determine the relative expression levels of some key genes related to growth and immune function, namely, insulin-like growth factor 1 (IGF-1), tumor necrosis factor-alpha (TNF-α), and interleukin-1 beta (IL-1β).

Amplification was carried out on a StepOne™ Real-Time PCR System (Applied Biosystems, Foster City, CA, USA) using the gene-specific primers listed in Table 2. The thermal cycling protocol comprised initial denaturation at 95 °C for 30 s, followed by 40 cycles of denaturation at 95 °C for 10 s and annealing at 58 °C for IGF-1 or at 60 °C for TNF-α and IL-1β for 30 s. Each 20 µL reaction contained 10 µL of SensiFAST™ SYBR Green Master Mix (Bioline, UK), 2 µL of cDNA template, 0.5 µL each of forward and reverse primers, and nuclease-free water to adjust the final volume. The assays were performed in duplicate to confirm the reproducibility of the data. Glyceraldehyde-3-phosphate dehydrogenase (GAPDH) was used as the reference gene, and relative expression was calculated the 2^^−ΔΔCt^ method^33^.

**Table 2:** Sequences of Primers used for the qRT-PCR.

Gene^1^	Primer	Reference
**GAPDH**	F: 5’-GCCGCTTCTTCTCGTGCAG-3’ R:5’ATGGATCATTGATGGCGACAACAT-3’	^34^
**IGF-1**	F: AGGAGGCTGGAGATGTACTG R: AAATGTACTTCCTTCTGAGTCT	^35^
**TNF-α**	F: 5’-CTGCACTTCAGGGTGATCG-3’ R: 5’-CTACGTGGGCTAGAGGCTTG-3’	^34^
**IL-1β**	F: 5’-TTGAAGAAGAACCCGTCCTCTG-3’ R: 5’-CTCATACGTGCCAGACAACACC-3’	^34^

^1^GAPDH housekeeping gene, insulin-like growth factor 1 (IGF-1); TNF-α: Tumor necrosis factor α; and IL-1β: Interleukin 1β.

### Cecal histomorphology

Three rabbits in each group were euthanized for histomorphological investigation. Histological specimens were taken immediately from the caeca, washed with normal saline, fixed in 10% neutral buffered formalin, dehydrated in an ascending series of alcohol (70–100%), cleared in xylene, embedded in paraffin and cut into sections of 4 μm thickness. The sections were then stained with hematoxylin and eosin (H&E) as described previously^36^. The stained sections were examined via a computerized light microscope (Leica DM 3000 LED). Quantitative measurements, including the height and width of the mucosal folds or villi as well as the cryptal depth and width, were conducted via morphometric software from Leica application suite, version 4.0 (LAS V4.0). Five random H&E-stained sections were evaluated for each rabbit by a single investigator. The mean values for all the morphometric measurements were statistically analyzed.

### Statistical analysis

The SPSS Computer Program (version 26.0; IBM Corp., Armonk, NY, USA) was used for statistical analysis of growth, biochemical and hematological parameters. Data analysis was performed via the general linear model (GLM) procedure. Post hoc comparisons were conducted via Tukey’s test to identify significant differences among group means. All the results are presented as the mean ± standard error of the mean (SEM), with statistically significant at (*P* < 0.05), whereas values of (*P* > 0.05) were considered non statistically significant. The statistical model that was used was as follows: *Yij = µ + Ti + eij*, where Yij represents the value that was noticed from the relevant treatment, µ denotes the recorded mean for the relevant treatment, Ti signifies the treatment impact, and eij indicates the error associated with the individual observation. Moreover, statistical analysis of the gene expression data was also performed via one-way ANOVA and Tukey’s post hoc test for multiple comparisons via GraphPad Prism 5 (San Diego, CA, USA).

## Results

### Proximate composition of dried guava pomace

The proximate chemical analysis of dried guava pomace is presented in Table 3. The analyzed DGP contained a dry matter content of 94.35%. On a dry matter basis, crude protein (CP), ether extract (EE), crude fiber (CF), total ash (TA), and nitrogen-free extract (NFE) accounted for 7.53%, 18.93%, 59.21%, 1.27%, and 7.41%, respectively^37^. Digestible energy (DE) content was calculated to be 2012 kcal/kg using to the equation of Fekete and Gippert^37^.

**Table 3:** Proximate composition of guava pomace (*Psidium guajava* L.) on DM basis.

Component	Value
Dry matter (DM, %)	94.35
Crude protein (CP, %)	7.53
Ether extract (EE, %)	18.93
Crude fiber (CF, %)	59.21
Ash (%)	1.27
Nitrogen-free extract (NFE, %)	7.41
Digestible energy (DE, kcal/kg) *	2012

DE was calculated according to the equation of^37^. DE (kcal/kg DM) = 4253–32.6 × (CF %) – 144.4 × (Ash %).

### Growth performance

Growth performance parameters are summarized in Table 4. NWZ rabbits fed diets containing DGP, either alone or in combination with prebiotics or organic acids (T1, T2, and T3), showed higher significant difference in FBW, and ADG compared with rabbits fed the control diet (*P* < 0.01). Similarly, DFI and TFI showed lower significant difference in the supplemented groups than in the control group (*P* = 0.012). However, FCR and PI values were significantly improved in rabbits receiving DGP-based diets compared with the control group (*P* < 0.01).

**Table 4:** Production parameters of New Zealand White rabbits in response to dietary feed additives from days 28–98.

Traits	Treatments^1^				SEM	*P*-value
	CON	T1	T2	T3		
IW (g)	434.58	425.42	434.17	450.42	12.47	0.913
FBW (g)	1949.17^b^	2196.25^a^	2227.50^a^	2235.00^a^	24.58	**< 0.001**
FWG (g)	1514.58^b^	1770.83^a^	1793.33^a^	1784.58^a^	20.62	**< 0.001**
ADG (g)	24.04^b^	28.11^a^	28.47^a^	28.33^a^	0.33	**< 0.001**
TFI (g)	7075.54^a^	6351.60^b^	6253.95^b^	6455.26^b^	91.55	**0.012**
DFI (g)	112.31^a^	100.82^b^	99.27^b^	102.46^b^	1.45	**0.012**
FCR (g feed/g gain)	4.69^a^	3.60^b^	3.50^b^	3.64^b^	0.06	**< 0.001**
PI	41.87^b^	61.72^a^	64.91^a^	62.07^a^	1.24	**< 0.001**

^a, b^ Means within the same a row bearing different superscripts are significantly different (***P*** **< 0.05**). ^1^**CON**, control basal diet; **T1**, basal diet supplemented with 20% DGP; **T2**: basal diet supplemented with 20% DGP and 1.0% prebiotic source of MOS and β-glucans; **T3**: basal diet supplemented with 20% DGP and 1.0% of a mixture of organic acids, propionate, binder and additional supportive components IW, initial body weight; FBW, final body weight; FWG, final weight gain; ADG, average daily gain; TFI, total feed intake; DFI, daily feed intake; FCR, feed conversion ratio; PI, performance index. SEM: Pooled standard error of the mean.

### Biochemical and hematological parameters

Serum biochemical and hematological parameters are shown in Table 5. Dietary treatments (T1-T3) were not significantly affected (*P* > 0.05) for total protein, ALB, GLOB, urea, CR, ALT and AST.

**Table 5:** Biochemical and hematological parameters of New Zealand White rabbits in response to dietary feed additives.

Parameter	Treatments^1^				SEM	*P*-value
	CON	T1	T2	T3		
Total protein (g/dl)	7.50	6.07	7.77	7.20	0.55	0.721
Albumin (g/dl)	3.17	2.80	2.83	2.90	0.08	0.370
Globulin (g/dl)	4.33	3.27	4.93	4.30	0.59	0.799
Urea (mg/dl)	163.57	153.40	158.57	154.40	4.21	0.823
Creatinine (mg/dl)	1.29	1.23	1.02	0.94	0.09	0.581
ALT (U/L)	47.70	37.20	52.60	35.43	2.75	0.159
AST (U/L)	52.60	35.43	50.00	40.13	2.74	0.168
Hb (g/dl)	10.88 ^b^	11.57 ^b^	11.65 ^b^	12.91 ^a^	0.16	**0.002**
RBCs (* 10^6^/µL)	4.38	4.01	4.07	4.65	0.11	0.145
PCV (%)	33.67 ^b^	35.67 ^ab^	35.83 ^ab^	40.33 ^a^	0.62	**0.007**
PLT (* 10^3^/µL)	244.67	236.33	310.00	236.17	16.30	0.229
WBCs (* 10^3^/µL)	5.27	5.70	7.58	6.77	0.43	0.252
N %	52.00	55.00	59.17	55.50	2.19	0.694
L %	42.33	40.00	34.17	38	1.87	0.456
M %	5.67	5.00	6.67	6.50	0.58	0.733

^a, b^ Means within the same row bearing different superscripts are significantly different (***P*** **< 0.05**). ^1^**CON**, control basal diet; **T1**, basal diet supplemented with 20% DGP; **T2**: basal diet supplemented with 20% DGP and 1.0% prebiotic source of MOS and β-glucans; **T3**: basal diet supplemented with 20% DGP and 1.0% of a mixture of organic acids, propionate, binder and additional supportive components. ALT, alanine aminotransferase; AST, aspartate aminotransferase; Hb, hemoglobin; PCV, packed cell volume; PLT, platelets; N, neutrophils; L, lymphocytes; M, monocytes. SEM: Pooled standard error of the mean.

The results of hematological indices, Hb concentration was significantly higher in the T2 group compared with the control group (*P* < 0.05), while PCV was substantially greater in the T3 group (*P* < 0.05). However, other parameters such as RBCs count, WBCs count, platelet count, and differential leukocyte profiles did not differ significantly among the experimental groups (*P* > 0.05).

### Gene expression in cecal and muscle tissues

**Fig. 1:**
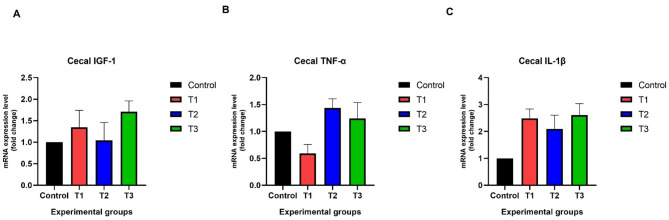
Relative mRNA expression levels of IGF-1 **(A)**, TNF-α **(B)**, and IL1-β **(C)** in the cecum of rabbits fed diets supplemented with 20% dried guava pomace (DGP) (T1), 20% DGP with 1.0% prebiotic (T2), or 20% DGP with 1.0% organic acids additive (T3). The data are expressed as mean ± standard error. No significant differences were detected among experimental groups at *P* > 0.05.

**Fig. 2:**
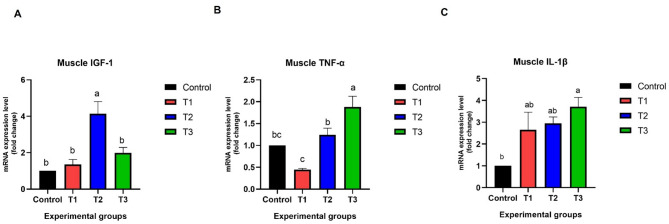
Relative mRNA expression levels of IGF-1 **(A)**, TNF-α **(B)**, and IL-1β **(C)** in the muscle tissues of rabbits fed diets supplemented with 20% dried guava pomace (DGP) (T1), 20% DGP with 1.0% prebiotic (T2), or 20% DGP with 1.0% organic acids additive (T3). The data are expressed as mean ± standard error. Bars with different superscript letters (a, b) differ significantly among treatments (*P* < 0.05).

The mRNA expression levels of IGF-1, TNF-α, and IL-1 β in both cecal and muscle tissues of NWZ rabbits are shown in Figs. 1 and 2.

### IGF-1 expression

Cecal IGF-1 expression was not significantly affected among experimental groups (*P* > 0.05; Fig. 1A), In contrast, muscle IGF-1 expression differed significantly among treatments (*P* < 0.05; Fig. 2A), with higher expression noted in DGP plus prebiotics supplemented group (T2) compared with the control.

### TNF-α expression

Cecal TNF-α expression was not affected significantly among groups (*P* > 0.05; Fig. 1B). However, muscle TNF-α expression was significantly affected by dietary treatments (*P* < 0.05; Fig. 2B), with lower expression recorded in the T1 group and higher expression in the T2 and T3 groups compared with the control.

### IL-1β expression

Cecal IL-1β expression was not affected significantly among different dietary treatments (*P* > 0.05; Fig. 1C). However, muscle IL-1β expression was substantially higher in all supplemented groups especially T3 compared with the control (*P* < 0.05; Fig. 2C).

### Cecal histomorphology

**Fig. 3:**
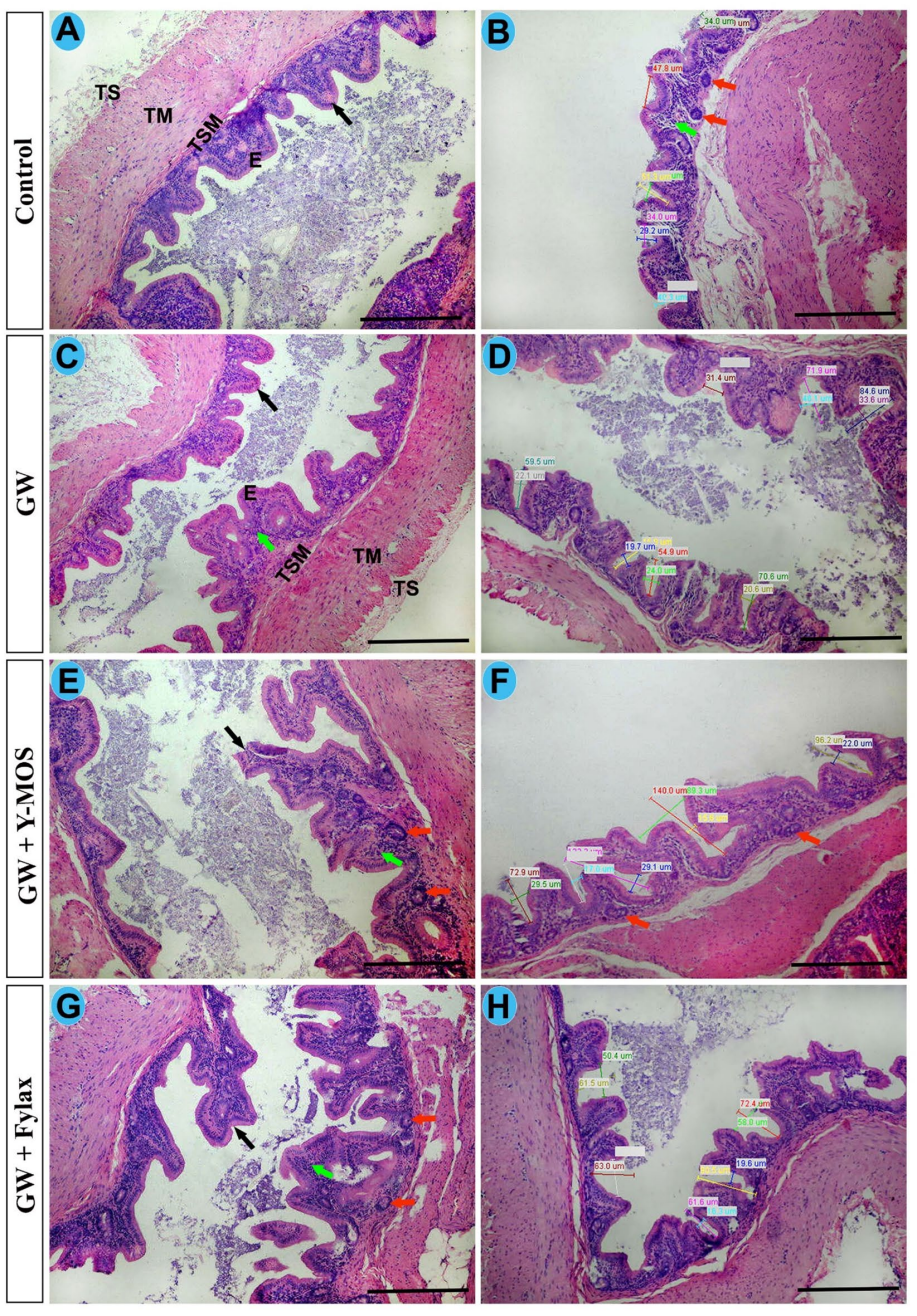
Photomicrograph of the cecum of the rabbits in the control group (**A**,** B**), 20% DGP group (**C**,** D**), 20% DGP with 1.0% prebiotic group (**E**,** F**) and 20% DGP with 1.0% organic acids additive group (**G**,** H**) showing mucosal folds or villi (black arrows) lined by simple columnar epithelium (E) and numerous scattered lymphocytes (green arrows) in the lamina propria as well as lymphoid tissue in the germinal center (red arrows) in the tunica submucosa (TSM). Notably, the greatest number of mucosal folds or villi and greatest crypt depth between the mucosal folds were found in the Y-Mos group compared with those in the control and other groups. Epithelium (E), Tunica submucosa (TSM), Tunica muscularis (TM), Tunica serosa (TS). H&E, Scale bar: 30 μm (A-H).

Histologically, the tunica mucosa of the rabbit cecum possessed numerous folds lined by simple columnar epithelium (Fig. 3A). These mucosal folds had different sizes and were separated by crypts. In the lamina propria mucosae, there were numerous scattered lymphocytes as well as lymphoid nodules or tissues in the form of germinal centers in the tunica submucosa (Fig. 3B). The tunica muscularis had an inner circular and outer longitudinal smooth muscle fiber layer (Fig. 3A).

The histological outcomes revealed that DGP supplementation increased the cecal mucosal fold height in NZW rabbits, but the greatest improvement found when fortified with the prebiotics plus DGP (T2) compared with the control and other groups (Figs. 3A, C, E & G), accompanied by a greater epithelial mass, greater surface area, and better nutrient uptake. Compared with the control and other groups, the T2 group also presented increased crypt numbers and depths (Figs. 3B, D, F & H).

## Discussion

This study examined how the impact of dietary inclusion of DGP, either alone or combined with a prebiotic source of mannan oligosaccharides and β-glucans or an organic acid blend, influences growth performance, selected blood parameters, tissue specific gene expression and cecal histomorphology in NZW rabbits.

Guava byproducts have a high content of dietary fibers, and secondary metabolites, which may exhibit antioxidative, immunomodulatory, and antibacterial properties^38^. As Egypt is one of the world’s major guava producers, the use of guava by-products for livestock feed offers a practical solution for waste reduction and improvement of feed quality. Nevertheless, the integration of guava waste or guava pomace in rabbit nutrition has received little research interest. Previous investigations, such as those of Morsy et al.^39^, suggested that guava leaf extract (GLE) might enhance growth and modify some blood biochemical properties in APRI rabbits.

The novelty of this study is about the combined effects of guava pomace with prebiotic and organic acids on the growth, selected blood parameters, cecal morphology and the mRNA expression of some growth and immune related genes in fattening rabbits.

DGP may provide dietary fiber and bioactive compounds contributing to the observed performance responses, antioxidant activity, better growth, healthier blood profiles, and modulated selected immune-related gene expression in cecal and muscle tissue which reinforced by combination with prebiotics and organic acids. These advantages come from the anti-inflammatory effects of the guava pomace working in tandem with mannan-oligosaccharides and β-glucan as well as organic acids to improve gut health. While using “waste” products for precision nutrition is a promising strategy, the next step is determining the most cost-effective inclusion levels and observing the long-term effects on commercial farms.

### Proximate analysis

The guava pomace investigated in the current study had a CP content of 7.53%. Even though this figure was a bit lower, it still falls inside the range indicated in earlier studies^40–42^, whose CP values between 8.60% and 10.09%. However, EE concentration was 18.93%, which is dramatically higher than the range of 9.69% to 11.68% reported previously by the same researchers. Variations in the fruit kept during processing or alterations in the fat composition might explain this greater EE level. The current results also revealed a value of 59.21% for CF, consistent with the findings of Lira et al.^41^, whose CF percentages ranged from 56.01% to 60.08%. These outcomes; however, are not corresponding with the values of CF content stated previously^40,42^, which ranged from 39.5% to 46.88%. Such variations may be attributed to differences in pomace structure, specifically the quantities of peel, pulp, and seeds remaining during juice extraction. Also, the ash concentration was 1.27%, which is lower than previously reported values, ranging from 2.21% to 2.52%^40,42^. The variations in proximate composition reported in different studies may be attributed to several interacting factors, including differences in guava cultivar, local climate and soil conditions, the stage of fruit maturity at harvest, and processing methods especially drying temperature and time. Collectively, these factors highlight the need for region-specific compositional studies to accurately determine the nutritional worth of guava pomace utilized as a feed component.

### Growth performance

Compared with those in the control group, the growth performance parameters of rabbits fed diets with DGP alone or in combination with prebiotics or organic acids were superior. The present results indicated that the inclusion of 20% DGP in rabbit diets could enhance growth performance and feed efficiency, which is likely due to the beneficial effects of some bioactive compounds and fiber on nutrient utilization. Moreover, the growth performance parameters were improved upon supplementation of DGP with prebiotics and organic acids, likely due to beneficial impacts on gut health, intestinal microflora modulation, and nutrient utilization. These findings are in agreement with those of Abdelghani et al.^43^, who reported that dietary supplementation with GLE at 15 and 20 mg/kg significantly increased final body weight, daily weight gain, and feed intake compared with those of non-supplemented controls. This improved growth performance may be attributed to better nutrient digestibility, which is likely influenced by bioactive compounds such as gallic acid, ferulic acid, catechin, and caffeic acid, which help maintain gut integrity and support nutrient absorption^44^.

A growing body of literature has also provided evidence of the beneficial effects of guava-derived byproducts, especially guava leaf meals, on the productivity of chickens and rabbits. For example, scholars^24,45,46^ revealed considerable improvements in growth performance, which were mostly related to the increased digestibility and antibacterial characteristics of GLEs. These characteristics are essential for preserving the balance of gut microbes and avoiding dysbiosis, which frequently impairs feed intake and nutrient absorption^47,48^. Additionally, the secondary metabolites found in GLE, including gallic acid, p-coumaric acid, kaempferol, and quercetin, have antimicrobial and antioxidant properties that support intestinal health and, consequently, overall growth performance^49–51^. In accordance with these earlier publications, the present data revealed that the combination of DGP and prebiotics generated the most beneficial growth outcomes in NZW rabbits. This improvement could be partially explained by the increased digestibility and absorption of nutrients offered by β-glucan supplementation^52,53^. Greater villus height and other improvements in gut morphology have been linked to improved growth efficiency and nutrient intake^54^. Additional studies have further substantiated the importance of prebiotics in supporting gut health by increasing villus length and stimulating immune responses^3,55,56^. Earlier research by Mourão et al.^57^ revealed that supplementing rabbit diets with MOS at 1 g/kg significantly increased the total volatile fatty acid (VFA) content while concurrently lowering the cecal pH compared with those in both the untreated control and antibiotic-fed groups. In addition to supporting its prebiotic ability to improve gut health by regulating the intestinal microbiota, MOSs have been demonstrated to reduce microbial loads in the gastrointestinal tract, such as populations of *coliforms* and *enterococci* in the cecum and total bacterial counts in the ileum. Similarly, the introduction of organic acids into animal diets has been well demonstrated to improve intestinal health and performance across numerous species, including chickens. These benefits are mostly related to the loss of dietary buffering capacity and gut pH, which results in an unfavorable environment for acid-sensitive bacteria^58^. Moreover, organic acid supplementation has been related to better nutrient digestibility and enhanced microbial fermentation. For example, the study of Ma et al.^59^ reported higher cecal formate levels in chickens supplemented with mixed organic acids, likely due to the inclusion of formate as a primary component of the additive. The concentrations of acetate, butyrate, and isobutyrate in the cecum of broiler chickens were verified by parallel findings by Aljumaah et al.^19^. These results were attributed to the stimulation of acid-producing bacterial populations in response to the acidified intestinal environment.

The presence of these short chain fatty acids (SCFAs) particularly acetate and butyrate offer many physiological benefits. Both molecules serve as essential energy substrates for colonocytes and are involved in the activation of glyoxylate cycle enzymes that promote microbial access to alternative carbon sources^60,61^. The study of Maj et al.^7^ further noted that dietary organic acids can stimulate SCFA production, thereby leading to improved intestinal barrier function, nutrient absorption, and general health status in monogastric animals. Therefore, the combined use of guava pomace with either MOS-based prebiotics or organic acids may provide considerable advantages in terms of intestinal fermentation, microbial balance, and host nutrient utilization, eventually supporting increased gut health in growing rabbits. These findings collectively highlight the potential of DGP as a nutritional strategy to improve gut health and growth performance in growing rabbits, especially when it is fortified with prebiotics source from mannan-oligosaccharides and β-glucan or organic acids.

### Biochemical and hematological indices

The health and physiological condition of rabbits can be assessed by evaluating blood hematology. When a new feed additive is used, it is critical to assess changes in serum metabolites and blood profiles to confirm the safety of the additive^43^. In the present study, the measured serum biochemical parameters, including total protein, albumin, urea, creatinine, ALT, and AST, were not significantly affected (*P* > 0.05) by the different dietary treatments. However, rabbits supplemented with DGP + prebiotics presented numerically higher concentrations of total protein and globulin than did the control group. These results could be attributed to enhanced immune system activation and protein metabolism, as well as improved gut health and food absorption. These findings correlate with those of Mahmoud et al.^62^, who reported increased plasma protein levels following the inclusion of 1% dried guava leaves in broiler diets. However, the results of Morsy et al.^39^ reported no discernible effects of guava extract on total plasma protein, suggesting that the form and dosage of the guava product utilized may affect the variability. Similar non-discernible effects of 10–20% dry tomato pomace on total proteins and albumin were reported in growing rabbits^63^.

In terms of hematological indicators, the addition of DGP with organic acids (T3) significantly increased the packed cell volume and hemoglobin concentration compared with those of the control, indicating enhanced erythropoiesis and systemic oxygen delivery. Greater nutritional absorption and physiological resilience are facilitated by the role of organic acids in boosting antioxidant defense systems, improving gastrointestinal function, and reducing subclinical inflammation. These findings are in line with earlier studies that revealed the positive impacts of organic acids on intestinal function, immunological regulation, and performance^64–68^.

Guava leaf extract, a well-known source of bioactive polyphenols, possesses antioxidant and immunomodulatory properties. For example, a study by Abdelghani et al.^43^ reported that rabbits receiving GLE-supplemented diets presented significant increases in immunoglobulin G and A levels, accompanied by increased levels of antioxidant indicators such as superoxide dismutase and total antioxidant capacity. Furthermore, malondialdehyde and reactive oxygen species, two indicators of oxidative stress, decreased in tandem with these benefits, suggesting that GLE has strong antioxidative properties, most likely due to its phenolic components. The immunostimulatory properties of GLE have also been corroborated^46,69^, with a potential link to its high vitamin C content, which may improve disease resistance and stress tolerance. Consistently, dietary supplementation of prebiotic (Bio-Mos^®^, mannoligosacchride), and probiotic (Bio-Plus^®^ 2B, Bacillus subtilis and Bacillus licheniformis) and their mixture improves cell-mediated immune response, liver and kidney functions, decreased the mortality and improved the adverse clinical signs and post mortem lesions in in rabbits experimentally infected with *P. multocida*^70^. The results of complete blood count (CBC) also revealed a significant increase (*P* < 0.05) in the hemoglobin concentration and packed cell volume in rabbits fed the DGP + organic acids diet (T3) compared with those in the controls, reinforcing its role in improving the oxygen-carrying capacity and red cell mass. While red blood cell counts were not significantly affected, platelet counts varied significantly between the groups. Although WBC counts were not significantly different, both the DGP + prebiotics and DGP + organic acids groups (T2 and T3) presented numerically higher values, possibly indicating modulated selected immune-related gene expression in cecal and muscle tissue. These patterns are consistent with previous research^39^, which also revealed that supplementation with guava increased the Hb, PCV, RBC, and PLT values while keeping all of these values within physiological ranges for rabbits^71^. Guava leaves may also have a beneficial effect on the immune state of broilers, according to Ali and Shamsuzzaman^72^, possibly because guava leaves contain flavonoids that have antibacterial functions, such as *Staphylococcus aureus*. Moreover, the elevation in PCV values observed in this study may reflect better hematopoiesis and overall health, whereas declines in PCV often signify hematological stress or toxicity^73^. Guava lectins reduce *Escherichia coli* adherence to the intestinal lining, thereby lowering infection risk and mortality rates^39,74^. A potential decline in the WBC count in non-stressed rabbits further supports the concept of better welfare, as WBC counts frequently increase by 15–30% during stress^75^.

Additional evidence^43^ confirmed the lipid-lowering activity of GLE, which demonstrated peak reductions at 20 mg/kg and drastically lowered the serum levels of triglycerides, total cholesterol, LDL, HDL, and VLDL in a dose-dependent manner. The bioactive compound Asiatic acid—a triterpenoid in guava leaves—has been found to exert hepatoprotective and anti-inflammatory effects, lowering lipid accumulation and hepatic inflammation^76,77^. In line with these findings, reductions in plasma ALT and AST levels in GLE-treated rabbits suggest preserved liver integrity and reduced oxidative damage. Previous investigations further suggested the hypolipidemic action of guava extracts. Both investigators^78,79^ reported substantial decreases in plasma lipids, suggesting guava’s potential in nutritional therapy for hyperlipidemia in livestock. Moreover, Morsy et al.^39^ reported that GLE at 2 and 3 mL/kg successfully decreased triglycerides, total cholesterol, and LDL while increasing HDL. Similarly, Crespo and Esteve-Garcia^80^ reported that *Psidium guajava* extract might enhance lipid profiles by lowering triglyceride and LDL levels while increasing HDL levels. These benefits may occur from changes in hepatic lipogenesis, resulting in decreased lipid release into the bloodstream^81^.

### Expression of IGF-1, TNF-α and IL-1β genes

In the current study, feeding DGP to rabbits’ diet—either alone or combined with prebiotic or organic acid additives—altered the expression of some key genes related to growth and immunity in a tissue-dependent manner. The pronounced effect was observed in muscle, where IGF-1 mRNA increased sharply in the group given DGP plus prebiotics (T2). These results showed that muscle IGF-1 has a possible local anabolic signaling effect related to guava’s bioactive compounds and the prebiotic blend, a link consistent with the role of IGF-1 in muscle protein deposition and growth promotion^24^.

Cecal IGF-1 was not significantly affected, however, the higher numerical values in the DGP plus organic acids group (T3) may suggest localized trophic effects that promote mucosal development. This is consistent with the capacity of guava polyphenols and vitamin C to strengthen mucosal immunity and the gut structure^82^.

The proinflammatory marker TNF-α acts differently across tissues. In the cecum, the levels remained statistically stable, although those in the combined-supplement groups (T2, T3) tended to increase. In muscle, however, DGP alone (T1) produced a marked decrease in TNF-α, suggesting an anti-inflammatory influence of the pomace itself. The higher values at T2 and T3 could reflect the local inflammatory modulation of the additive combinations, owing to the dual nature of TNF-α in both defense and inflammation^83^. However, IL-1β expression tended to increase in the cecal and muscular tissues with variability in the local inflammatory modulation across treatments.

Guava is a rich source of bioactive compounds, including tannins, flavonoids, and vitamin C, which have been associated with antimicrobial, antioxidant, and immune-modulating activities. Daing et al.^46^ demonstrated that tannin-rich guava leaf extracts enhance immune response in broilers chickens, Similarly, others^69,84^ reported improved stress tolerance and growth performance when guava extracts were added to animal diets. Prebiotic compounds, such as MOS and isomalto-oligosaccharides, have also been reported to modulate gene expression which improve growth and immune function. According to Abd El-Aziz et al.^3^, MOS supplementation decreased intestinal IL-6 while increasing hepatic GPx1, IGF-1, and SOD1 activity, in addition to upregulating IGF-1 receptor expression in muscle tissue. In contrast, the expression of certain genes such as FAS, iNOS, and IL-6 in both muscle and spleen remained unchanged, suggesting the selective responsiveness of specific molecular targets to dietary intervention.

In addition, organic acids exert their individual modulatory effects. According to Lin et al.^85^, mixed acids decrease IL-6, tend to decrease IL-1β, and increase ZO-1 in the jejunal mucosa of rabbits; all these results are associated with improved barrier function and cytokine levels. In conclusion, the results showed that DGP diets especially in combination with functional additives such as prebiotics or organic acids can modulate anabolic gene networks and regulate some immune-related gene expression among selected tissues in response to dietary supplements. The growth and modulated selected immune-related gene expression in rabbits are likely due to these molecular shifts, which suggest that plant-based feed additives could be one tool for making rabbit production more sustainable.

### Cecal histomorphology

Interestingly, DGP improved nutrient digestibility by increasing the height of the cecal mucosal folds. However, the average height of the mucosal folds was significantly greater in the group fed a diet supplemented with a combination of DGP + prebiotics than in the control and other groups. Thus, the epithelial cell mass and surface area increased, which improved nutrient absorption. Moreover, the number of crypts was greater in the group fed a diet supplemented with DGP + prebiotics than in the control and other groups. These results are in agreement with those of Omitoyin et al.^86^, who reported that the villus height and width were increased in *Oreochromis niloticus* fed a diet supplemented with Guava extract. These results were consistent with those previously described^87–89^, where supplementation with a combination of DGP + organic acids in the diet had a proliferative effect on the intestinal villi, increasing their surface area and enhancing gastrointestinal cell proliferation. According to Mourão et al.^57^, the effect of MOS may be related to improvements in intestinal morphology, increased length of ileal villi, and digestibility of nutrients. First, MOS works by attaching itself to unwanted microbes, blocking their ability to bind to the intestinal mucosa and compete with its sugar receptors. Second, MOS activates the immune response in the intestines. According to Falcão-e-Cunha et al.^12^, this action stimulates the proliferation of beneficial microorganisms, enhances meal digestion and absorption, and finally improves performance. In addition, the use of guava waste in dietary fiber can modulate the composition of the intestinal microbiota and fermentation activity of beneficial bacteria (e.g., *Bifidobacterium* and *Lactobacillus*)^90,91^, which increase the production of short-chain fatty acids that contribute to the maintenance of intestinal health^92^. Furthermore, enhanced villous architecture in supplemented rabbits provided clear evidence of improved intestinal health, reflecting a greater capacity for nutrient absorption. This observation is consistent with the findings of Wang et al.^44^, who reported comparable gut mucosal improvements following GLE supplementation in other livestock. By increasing villous height and maintaining epithelial integrity, as shown in the present study, GLE may help safeguard intestinal barrier function by reducing permeability and sustaining nutrient uptake during the critical postweaning phase.

## Conclusions

Under the present experimental conditions, 20% DGP, alone or combined with gut modulators such as prebiotics or organic acids, improved selected growth performance and cecal histomorphological traits and modulated muscle expression of the immune-related IGF-1, TNF-α and IL-1β genes in growing rabbits. This supplementation aims to leverage the combined benefits of dietary fibers, bioactive polyphenols, and gut-modulating additives to increase productive and physiological outcomes for feeding rabbits.

### Limitations of the study

Several limitations of the current study should be acknowledged in the following points:

- First, the absence of treatment groups receiving prebiotics or organic acids alone prevents evaluation of additive versus interaction effects.
- Second, although proximate composition of DGP was determined, phytochemical components such as phenolic compounds were not directly quantified, limiting the ability to link observed biological effects of specific bioactive constituents.
- Third, gene expression analyses were carried out on a limited number of samples and represent local tissue responses rather than systemic physiological effects.
- Fourth, cecal microbiota composition was not assessed, which would have strengthened interpretation of the histomorphological findings.
- Finally, this is the first study for using guava pomace, however, analysis of its bioactive composition and how it is different from GLE was not assessed.

## Data Availability

All the data are provided within the manuscript. The qPCR datasets generated and/or analyzed during the current study are available in the Zenodo repository with DOI: https://doi.org/10.5281/zenodo.20563005.

## Abbreviations

GP: Guava Pomace
DGP: Dried Guava Pomace
GLE: Guava Leaf Extract
MOS: Mannan-oligosaccharides
CP: Crude Protein
EE: Ether Extract
CF: Crude Fiber
NFE: Nitrogen-free extract
DE: Digestible Energy
ADG: Average Daily Gain
TFI: Total Feed Intake
DFI: Daily Feed Intake
FCR: Feed Conversion Ratio
PI: Performance Index
IGF-1: Insulin-like Growth Factor 1
TNF-α: Tumor Necrosis Factor alpha
IL-1β: Interleukin-1 beta
SCF: As short chain fatty acids
